# Fregoli Syndrome: An Underrecognized Risk Factor for Aggression in Treatment Settings

**DOI:** 10.1155/2011/351824

**Published:** 2011-07-14

**Authors:** Nauman Ashraf, Daniel Antonius, Arthur Sinkman, Karine Kleinhaus, Dolores Malaspina

**Affiliations:** Department of Psychiatry, New York University School of Medicine, New York, NY 10016, USA

## Abstract

Fregoli syndrome (FS) is commonly associated with verbal threats and aggressive behavior. We present a case of Fregoli syndrome leading to an assault. We discuss the possible underdiagnosis of FS, associated risk for aggression, and strategies to reduce that risk.

## 1. Introduction

The delusional misidentification syndromes (DMSs) include the Capgras delusion, Fregoli syndrome, the syndrome of Intermetamorphosis, and the syndrome of Subjective Doubles. This category of delusional syndromes is characterized by paranoia and hostility towards misidentified objects [1], with behaviors ranging from verbal threats to severe physical injuries. The Capgras delusion, the belief that people have been replaced by impostors, has been widely documented [2, 3], and these patients have displayed assaultive behavior, particularly towards close relatives [1, 4]. Less known is Fregoli syndrome, the delusional belief that a single persecutor is masquerading as several other people, whose appearances he or she assumes at different times (for further review see [5]). In hospital settings, patients with Fregoli syndrome often misidentify members of the treatment team (e.g., nurses, doctors, trainees, etc.) who work closely with the patients. This misidentification may result in assaultive behavior towards the staff. Unfortunately, however, violence in Fregoli patients has been understudied. Here, we present a case in which a patient with Fregoli syndrome assaulted a psychiatrist. We then offer a clinical framework in which to understand these events. Accurate assessment of the syndrome and potential risk factors for future violence can help clinicians minimize assault risks and provide optimal treatment.

## 2. The Case

Mr. D is a 54-year-old single male with a longstanding history of schizophrenia, paranoid type, who was brought to the emergency room after he became increasingly paranoid and made threatening comments to the staff at his residential facility. He is well known to the clinical staff in the hospital and has been hospitalized multiple times, mostly due to persecutory delusions. He has a distant history of substance abuse, but he has not used substances since his late teens. His urine toxicology on admission was negative. He was previously treated with diverse antipsychotic medications and mood stabilizers. Mr. D's medical history includes HIV, Hepatitis C, treated syphilis with no evidence of neurosyphilis, head trauma, hypertension, and alleged history of seizure disorder. His CD4 count was 122 with a viral load of less than 75 K. A contrast recent enhanced MRI indicated no significant brain abnormalities. Neuropsychological testing suggested evidence of mild dementia, possibly related to HIV. 

Mr. D lost both of his parents during childhood. He does not remember his father's passing, but he was told that his mother committed suicide by overdosing on medication. He was raised by foster parents and suffered both physical and mental abuses by the foster father. Though he had a history of angry and agitated behavior, before the present admission the patient had no known history of violent behavior. During the admission interview, Mr. D presented with various delusional beliefs: for example, a nurse at the residence was trying to poison him, and staff were responding to commands from external voices telling them to harass him. He also presented somatic delusions, in which the bones in his face were shattered and pus was coming out of his ears. Although he was restarted on Risperidone, he continued to form new delusions involving his family being in danger, his feeling that the staff members at the hospital were against him, and that peers on the unit were putting chemicals into his eyes.

Two weeks into his hospitalization, Mr. D's psychiatric condition continued to be unstable. One morning Mr. D asked the nursing staff that he wanted to see his doctor, who at that time was evaluating another patient in the examination room on the inpatient unit. Mr. D was told by the staff to wait and that his doctor would see him soon. When Mr. D did not get his doctor's immediate attention, he became angry, forced his way into the examination room, and attempted to punch the doctor. Later, upon questioning, the patient reported that he believed that his doctor was only masquerading as a doctor and that he was actually the nurse. He said that the same nurse gave him the wrong medication another night, and that she was taking on the appearance of the doctor to further harm him. The patient was subsequently diagnosed with Fregoli syndrome. This episode of Fregoli syndrome was brief, lasting about one day, and there were no prior reports in Mr. D's history of delusions of doubles.

## 3. Discussion

Fregoli syndrome was first described in 1933 [6], a decade after Capgras and Reboul-Lachaux described the first case of look-alike impostors [7, 8]. The syndrome is considered a rare neuropsychiatric condition commonly linked to schizophrenia, schizoaffective disorder, and other organic illnesses [8, 9]. The frequency of violence in Fregoli syndrome is unclear. Silva et al. [1] described 144 cases of patients who exhibited violence towards misidentified people; of these, only 6 had Fregoli syndrome, 86 had Capgras, and 22 had other diagnoses. The most common Axis I diagnosis in that sample was paranoid schizophrenia (59.8%). Our patient was also diagnosed with paranoid schizophrenia. 

Neurobiological research on DMS points to lesions in both frontal lobes and/or right hemispheres. Right hemispheric lesions have particularly been associated with Fregoli syndrome. Underactivity in the perirhinal cortex seems to be responsible for loss of familiarity in Capgras, whereas overactivity seems to account for hyperfamiliarity seen in the Fregoli, Intermetamorphosis, and Subjective Doubles syndromes [4]. Impaired connectivity between the right fusiform and right parahippocampal areas has also been implicated in deficits in visual memory recall, face recognition, and identification processes in these patients [10].

Mr. D exhibited several factors including schizophrenia, HIV, mild dementia, head trauma, history of syphilis, and possibly seizure disorder—which had a detrimental effect on his brain, and may have resulted in Fregoli syndrome. Christodoulou [11] described patients diagnosed with paranoid schizophrenia who developed Fregoli delusions many years after their diagnosis and only after organic brain damage. Others [5] have also documented the association between DMS and organic brain disease.

The link between early trauma and later violence is widely known [12]. Traumatic events in Mr. D's early upbringing in the form of losing his parents at an early age and subsequent abuse by his foster father elicited feelings of mistrust in others. This mistrust was evident when Mr. D became angry at his doctor, who in his mind was a nurse masquerading as his doctor. Mr. D also has a combination of three different delusions: persecutory, somatic, and misidentification. This is a special grouping of delusions [8] in which the misidentification delusions can be brief and fleeting in duration as opposed to long-term and fixed delusions. 

Could Mr. D's assaultive behavior have been prevented? Although hindsight is 20/20, Gabbard [13] has suggested two relevant case management principles with Fregoli patients: (a) avoid arousing further suspicion and (b) always encourage the patient to verbalize rather than to violently act out his anger. Based on these principles, further suspicion may have been avoided if the doctor had spoken to the patient before seeing other patients that morning. This may have shed light on Mr. D's anger and his suspiciousness towards the treatment team. Additionally, nursing and other clinical staff could have spent more time with the patient which perhaps would have given him a chance to vent his anger. Gabbard's two case management principles warrant further empirical research for validation. 

Predicting assaults by psychiatric patients is difficult, and members of clinical staff are often either the victims or the first responders [14]. Due to the nature of the delusion, patients with Fregoli syndrome may present a subgroup of patients who are of particularly high risk for violence. It is therefore important that this relatively uncommon delusional syndrome is recognized by clinicians in order to decrease the assault risk and to ensure better patient treatment.
